# Mechanical intestinal obstruction secondary to appendiceal mucinous cystadenoma: A case report and brief review

**DOI:** 10.1097/MD.0000000000006016

**Published:** 2017-02-03

**Authors:** Zheng-shui Xu, Wei Xu, Jia-qi Ying, Hua Cheng

**Affiliations:** Department of General Surgery, The Second Affiliated Hospital of Nanchang University, Nanchang, Jiangxi, China.

**Keywords:** appendiceal tumor, intestinal obstruction, mucinous cystadenoma

## Abstract

**Background:**

Appendiceal mucinous cystadenoma can present in various ways, and it is most commonly encountered incidentally during appendectomy, but mechanical intestinal obstruction secondary to an appendiceal mucocele has been rarely reported.

**Methods:**

We report a case of mechanical intestinal obstruction secondary to appendiceal mucinous cystadenoma. After nasogastric decompression and initial aggressive intravenous fluid resuscitation, an emergency operation was performed under the diagnosis of acute mechanical intestinal obstruction.

**Results:**

We performed an appendectomy and intraoperative enteral decompression without anastomoses. The pathologic examination (PE) revealed appendiceal mucinous cystadenoma. After the operation, the patient's recovery went smoothly, and the patient was discharged on the fifth postoperative day. No tumor recurrence was recorded over an 8 month follow-up period.

**Conclusion:**

Early operative intervention should be recommended to the patient with acute mechanical complete intestinal obstruction, especially the patient who had no previous abdominal surgery. And it is vital to discriminate benign and malignantappendiceal mucocel in determining the extent of surgery.

## 1 Introduction

Mucocele is an uncommon disease of the appendix. Appendiceal mucocele can present in diversified ways, but it is most commonly encountered incidentally during appendectomy.^[1]^ A case report of mucinous cystadenoma, a particular type of mucocele, at the tip of the appendix that led to a mechanical intestinal obstruction has rarely been described in the literature. Here, we present a case report describing a 76-year-old man who was diagnosed with mechanical complete intestinal obstruction secondary to appendiceal mucinous cystadenoma.

## 2 Case report

**Figure 1:**
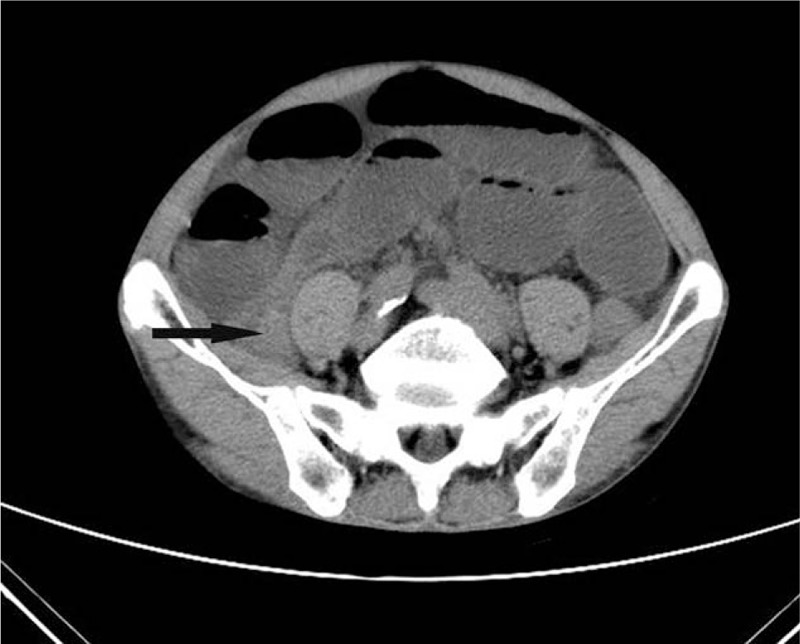
A CT scout of the abdomen revealed dilated small bowel loops with air-fluid levels suggestive of a small bowel obstruction. CT = computed tomography.

**Figure 2:**
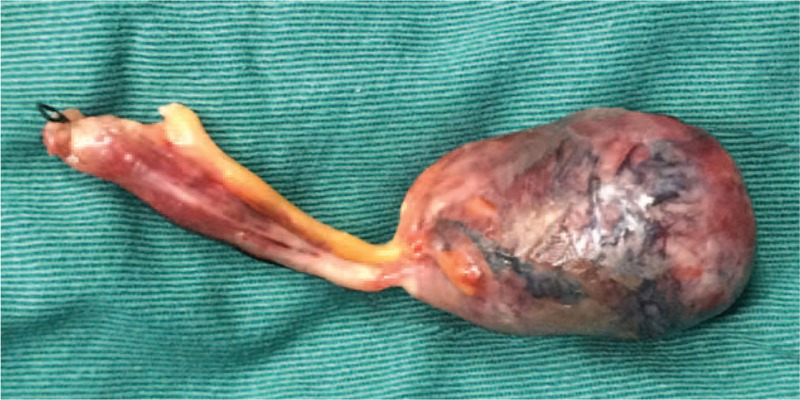
This is the appendiceal mucocele, about 4.0 × 2.5 × 2.5 cm.

**Figure 3:**
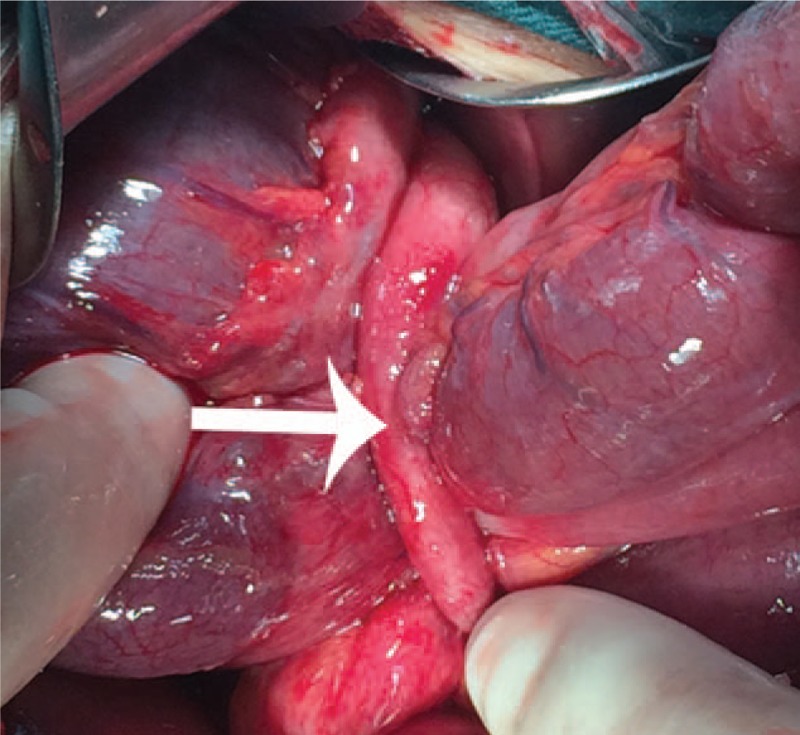
The mucocele has adhered to the right paracolic sulci like a loop, and the ileum was confined within the loop. The incarcerated ileum was between 2 and 60 cm from a leocecal valve drilled into the loop in a “U” shape. (Appendix is shown by arrow).

**Figure 4:**
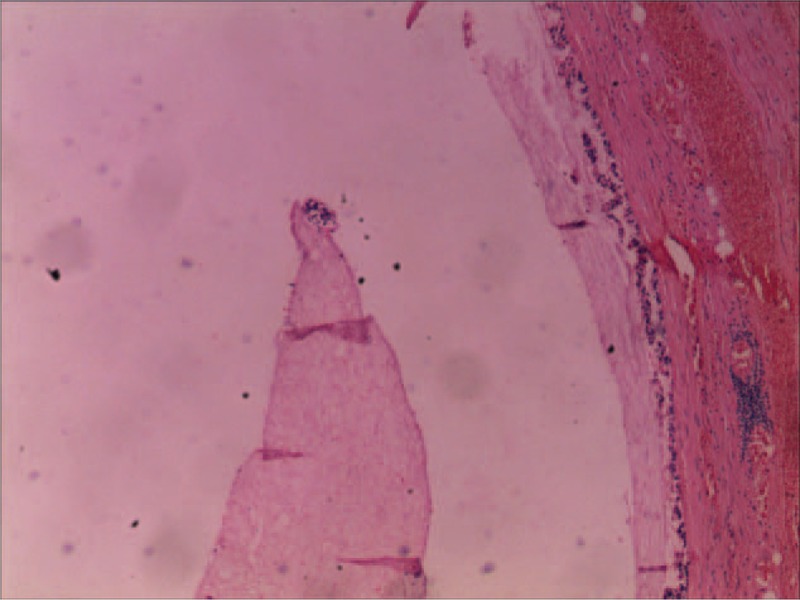
A 40 times magnification hematoxylin and eosin (H&E) stained for specimen of the removal appendiceal tumor reveals appendiceal mucinous cystadenoma.

A 76-year-old previously healthy man, who had no history of abdominal surgery, presented to our emergency room with a 38-hour history of intermittent abdominal pain, nausea, and the disruption of bowel movements. His abdomen was mildly distended and tympanic with diffuse slight tenderness, particularly in the periumbilical areas. He had sonorous bowel sounds. A computed tomography (CT) scan of the abdomen revealed dilated small bowel loops with air-fluid levels suggestive of a small bowel obstruction (Fig. 1). The patient's leukocyte count was 1890, with 92.1% neutrophils. After nasogastric decompression and initial aggressive intravenous fluid resuscitation, an emergency operation was performed under the diagnosis of acute mechanical complete intestinal obstruction. The patient underwent exploratory laparotomy that revealed dilated enteral loops with air-fluid content and uncomplicated mild ascites. In addition, there was an appendiceal mucocele present, measuring 4.0 × 2.5 × 2.5 cm, at the tip of the appendix (Fig. 2). The mucocele had adhered to the right paracolic sulci that formed a loop which the ileum was confined within. The incarcerated ileum was between 2 and 60 cm from a leocecal valve drilled into the loop in a “U” shape (Fig. 3). Because there was no intestinal necrosis, we performed an appendectomy and intraoperative enteral decompression without anastomoses. The pathologic examination revealed appendiceal mucinous cystadenoma (Fig. 4). After the operation, the patient's recovery went smoothly, and the patient was discharged on the tenth postoperative day. By telephone call following up, the patient indicated that he had been free of abdomen pain, nausea bloating, etc., and no tumor recurrence was recorded over an 8 months period.

## 3 Discussion

Appendiceal mucocele is a relatively rare disease,^[2]^ observed in 0.2% to 0.3% of appendectomies and 8% to 10% of appendiceal tumors.^[3]^ Appendiceal mucoceles are more frequent in women than in men, and are often discovered in patients older than 50 years.^[4]^ Mucocele is an uncommon pathology of the appendix characterized by a cystic dilation of the lumen as a result of an abnormal accumulation of mucus,^[1]^ and it usually results from either benign (cystadenoma) or malignant (cystadenocarcinoma) epithelial proliferation.^[5]^ Clinical manifestations of appendiceal mucoceles are quite diversified. 30% to 64% of patients present right lower quadrant pain, similar to the acute appendicitis, which is the most common manifestation.^[6–9]^ A quarter of patients are asymptomatic which are more frequently found in benign appendiceal mucocele, while appendiceal mucoceles are discovered incidentally during physical examinations (by palpation), at surgery or during imaging study for other problems.^[10,11]^ Other clinical manifestations mainly include intussusception, genitourinary symptoms, low gastrointestinal bleeding, and so on. ^[6,12]^ However a bowel obstruction secondary to an appendiceal mucocele has been rarely reported.^[13–16]^ And the early diagnosis is challenging. Imaging with CT has better apply to establish a preoperative diagnosis in similar cases.^[17]^

Although it would have been almost impossible to make the right diagnosis prior to the operation in this case, there are still many aspects that are worth reflecting on. First, the patient came into the emergency room with signs and symptoms of acute mechanical intestinal obstruction, and he had no previous abdominal surgery. Therefore, we almost could exclude the diagnosis of adhesive intestinal obstruction. The differential diagnosis at initial presentation included a colonic tumor, benign or malignant small intestinal tumors, and a stricture secondary to other conditions (e.g., enterocele, volvulus, intussusception, and so on), all of which often required an operation. Second, when we take the medical history of a patient with an unexplained intestinal obstruction, we should be more careful with the patient's past medical history and potential causes of the symptoms, these may help us make right diagnosis. In this case, we reinquiry the patient's case history on postoperative day, the patient had had a right lower quadrant abdominal intermittent slight chronic pain symptoms in the past 20 years, but there was no other influence in his work and daily life, so he did not care about the symptoms at all. Now if we integrated the medical history and CT, it seemed that we may have made the right diagnosis before operation. Third, we support the clinical application of total abdomen CT scanning in the case of an unexplained mechanism of abdominal obstruction. An abdominal CT is a rapid, simple, and effective means for diagnosing the location, cause, and degree of obstruction in cases of unexplained intestinal obstructions. It is particularly useful for orienting the clinician to the location of obstruction, and it would help surgeons decide where to make an incision, as an appropriately placed incision is crucial to the success of an operation.

So how did we determine the extent of surgery for the patient with appendiceal mucocele? It is vital to discriminate benign and malignant appendiceal mucocele in determining the extent of surgery. Appendectomy is appropriate therapy for unruptured benign appendiceal mucoceles, like this case. When benign appendiceal mucocele protrudes into cecal lumen, partial cecectomy may be curative. “If either cecal wall or ileum is invaded by tumor or adequate surgical margins cannot be secured, ileocecal section or right hemicolectomy may be required.”^[18]^ If malignancy cannot be exclusive, right hemicolectomy should be considered.^[1,18]^ In this case, we should exclude the possibility of the intestine necrosis; otherwise, we must resect the highly suspected necrotic intestinal tissue further. The key-point of surgical treatment is to make resection margin clear and keep the appendiceal mucocele intact. The prognosis for benign appendiceal mucocele following complete ablation is excellent and the 5-year survival rate is almost 100%.^[18]^ A patient with malignant mucocele, however, is much poorer, and the 5-year survival rate has association with the degree of extension of the tumor and varies between 30% and 80%.^[19]^

## 4 Conclusion

For acute mechanical complete intestinal obstruction, the patient's past medical history and potential causes of the symptoms should be paid attention to, and an abdomen CT is very necessary; Besides early operative intervention should be recommended to the patient with acute mechanical complete intestinal obstruction, especially the patient who had no previous abdominal surgery. Moreover, it is vital to discriminate benign and malignantappendiceal mucocel in determining the extent of surgery.
